# Predicting Wound Complications Following Total Knee Arthroplasty: Preoperative Inflammatory Indices and a Novel Risk Scale (TKA-PII)

**DOI:** 10.3390/diagnostics16152331

**Published:** 2026-07-25

**Authors:** Furkan Soy, Mustafa Can Yanık, Yasin Kozan, Ozan Pehlivan, Sancar Serbest

**Affiliations:** 1 Department of Orthopedics and Traumatology, Faculty of Medicine, Kırıkkale University, Kırıkkale 71450, Türkiye; mustafa_can_ynk@hotmail.com (M.C.Y.); pehlivanozan@gmail.com (O.P.); dr.sancarserbest@gmail.com (S.S.); 2 Department of Orthopedics and Traumatology, Akdağmadeni State Hospital, Yozgat 66300, Türkiye; yasinkozan.dr@gmail.com

**Keywords:** total knee arthroplasty, wound complications, SIRI, SII, PIIV, inflammation, risk assessment

## Abstract

**Background/Objectives**: This study investigated the value of several hemogram-derived biomarkers, including SIRI, SII, PIIV, NLR, MLR, and PLR, in predicting wound complications after total knee arthroplasty (TKA). **Methods**: This study involved 226 patients who underwent TKA between 2018 and 2024. Preoperative hematological parameters and their derived indices (NLR, MLR, PLR, PIIV, SII, and SIRI) were analyzed. To identify the parameters that predict the risk of complications, correlation analysis, ROC-curve analysis, and logistic regression analysis were performed. Additionally, a novel risk scale, entitled “Preoperative Inflammatory Index at Total Knee Arthroplasty (TKA-PII)”, was developed using factor analysis and a reliability test. **Results**: Postoperative wound complications were observed in 30 patients. NLR, MLR, PIIV, SII, and SIRI values were significantly higher in those who developed complications. Correlation analysis indicated a positive relationship between these inflammatory indices and the risk of developing complications. ROC-Curve analysis revealed that SIRI had the highest discriminative performance (area under curve (AUC) = 0.720), followed closely by PIIV (AUC = 0.716) and SII (AUC = 0.704) in predicting the complication risk. Logistic regression analysis identified that SIRI was the best predictor of postoperative wound complications (Odds Ratio = 2.277, *p* = 0.004). Additionally, the TKA-PII scale demonstrated acceptable validity and reliability (Bartlett’s test of sphericity = 0.609, Cronbach’s alpha = 0.647). **Conclusions**: This study’s findings suggest that preoperative hemogram-derived inflammatory indices, particularly SIRI, could be valuable markers for predicting the risk of wound complications after TKA. Furthermore, the newly developed TKA-PII scale could be used as an easy-to-use and cost-effective tool for preoperative risk assessment in clinical practice.

## 1. Introduction

Total knee arthroplasty (TKA) is a common procedure for treating end-stage knee osteoarthritis. Research indicates that early postoperative infection rates following TKA range from 0.5% to 5%, with 3.4% classified as superficial infections and 1.2% as deep infections. These wound complications can negatively impact joint function and result in significant postoperative pain. Additionally, they may lead to prolonged use of pain relief medications and antibiotics, extended hospital stays, and increased healthcare costs [1,2].

Currently, the erythrocyte sedimentation rate (ESR) and C-reactive protein (CRP) are widely used to diagnose and monitor periprosthetic joint infections. However, preoperative measurements of these markers have demonstrated low effectiveness in predicting postoperative wound complications. As a result, new hematological indices on this issue, such as pan-immune-inflammation value (PIIV), systemic immune-inflammation index (SII), systemic inflammation response index (SIRI), monocyte-to-lymphocyte ratio (MLR), neutrophil-to-lymphocyte ratio (NLR), and platelet-to-lymphocyte ratio (PLR), have gained attention in recent years. These indices are believed to provide a more comprehensive reflection of systemic inflammation [3,4,5,6,7,8,9,10]. However, studies investigating the efficacy of the preoperative values of these parameters in predicting postoperative complications remain highly limited [11,12].

Based on the available evidence demonstrating the association between systemic inflammatory indices and adverse postoperative outcomes, we hypothesized that patients with higher preoperative hemogram-derived inflammatory indices, particularly SIRI, SII, PIIV, NLR, MLR, and PLR, would have an increased risk of developing postoperative wound complications following primary total knee arthroplasty. We further hypothesized that these readily available biomarkers could provide a simple, cost-effective, and clinically useful approach for preoperative risk stratification and could serve as the basis for the development of a practical risk assessment scale.

## 2. Materials and Methods

This study was conducted with approval from the Non-Interventional Clinical Research Ethics Committee (Date: 28 May 2025; Meeting No: 2025/09; Decision No: 2025.05.36). The study adhered to the principles of the Declaration of Helsinki.

### 2.1. Patients

Patients who underwent primary TKA for end-stage knee osteoarthritis in the Department of Orthopedics and Traumatology between 2018 and 2024 were included in the study. They were then categorized into the following groups:

- Complication (-) group (this group included patients whose sutures were removed in the second postoperative week and who experienced no wound problems at the surgical incision line during the one-year follow-up, *n* = 196).
- Complication (+) group (this group consisted of the patients who required additional procedures beyond routine outpatient interventions or needed wound revision through simple surgical procedures at the surgical site starting from postoperative day one, *n* = 30).

Additionally, the patients were divided into two groups based on gender (male and female), and their data were then compared.

Patients with inadequate medical records, those diagnosed with periprosthetic joint infection and who had undergone revision knee surgery, those with less than one year of follow-up after the surgical procedure, patients with active infections or chronic inflammatory diseases, or a history of malignancy, patients with hematological disorders, patients using immunosuppressive therapy or corticosteroids preoperatively, and those who underwent revision TKA or additional surgical interventions during the follow-up period were excluded.

### 2.2. Materials

**Figure 1:**
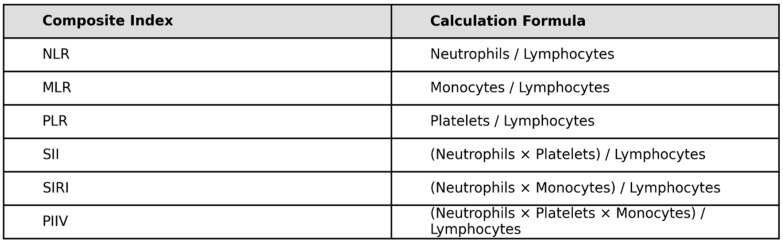
Hemogram-derived inflammatory indices along with their calculation formulas.

Patient age, gender, and operated site were recorded retrospectively using the hospital’s digital registry system. Furthermore, preoperative blood levels of neutrophils (reference range: 110–9600 μL), lymphocytes (reference range: 500–6000 μL), monocytes (reference range: 100–1400 μL), platelets (reference range: 150–500 × 10^3^ μL), serum blood urea nitrogen (BUN) (reference range: 17–43 mg/dL), creatinine (reference range: 0.84–1.24 mg/dL), alanine aminotransferase (ALT) (reference range: 5–41 U/L), aspartate aminotransferase (AST) (reference range: 5–40 U/L), HbA1c, total protein (reference range: 6.6–8.3 g/dL), and albumin (reference range: 3.5–5.2 g/dL) were recorded. Subsequently, the NLR was calculated as the neutrophil count divided by the lymphocyte count; the MLR was calculated as the monocyte count divided by the lymphocyte count; and the PLR was calculated as the platelet count divided by the lymphocyte count. Additionally, the PIIV was calculated by dividing the product of neutrophil, platelet, and monocyte counts by the lymphocyte count; the SII was calculated by dividing the product of platelet and neutrophil counts by the lymphocyte count; and the SIRI was calculated by dividing the product of neutrophil and monocyte counts by the lymphocyte count (Figure 1).

### 2.3. Surgery

All surgical procedures were performed by experienced surgeons using a standard technique. Prophylactic antibiotic therapy, first-generation cephalosporin (cefazolin), was routinely administered to all patients intravenously one hour before surgery. Clindamycin was provided as an alternative drug for patients with a penicillin allergy. If the duration of the surgery exceeded two hours, an additional dose of those antibiotics was administered. Standard perioperative care protocols, thromboprophylaxis, and a postoperative rehabilitation program were applied to all patients.

### 2.4. Outcome Measures

The primary endpoint of the study was the development of postoperative wound complications, including surgical site infection, prolonged wound drainage, wound dehiscence, or the necessity for additional surgery or medical intervention related to the wound.

### 2.5. Statistical Analysis

Statistical analyses were performed using SPSS version 20.0 (IBM Corp., Armonk, NY, USA). The Kolmogorov–Smirnov test was conducted to assess the normal distribution of the study results. The categorical data were analyzed using Pearson’s Chi-Square test. For non-parametric findings, the Mann–Whitney U test was used for comparisons. Spearman’s rho correlation test assessed the relationships between parameters. The ROC curve was used to identify the parameters that could predict the risk of complications. “Youden method” to find the optimum cut-off value [13]. This method is presented as follows: Cut-off value = Sensitivity + (Specificity − 1).

Additionally, binary logistic regression was employed to determine the best predictive markers for the risk of complications. The direction and strength of the associations between TKA-PII markers and the risk of complications development were quantified using Odds Ratios (ORs) and their corresponding 95% confidence intervals (95% CI) [14].

Finally, Factor analysis (i.e., Principal Component Analysis) and Reliability analysis were applied to evaluate the validity and reliability of the TKA-PII scale. A *p*-value of less than 0.05 was considered statistically significant [15,16].

## 3. Results

**Figure 2:**
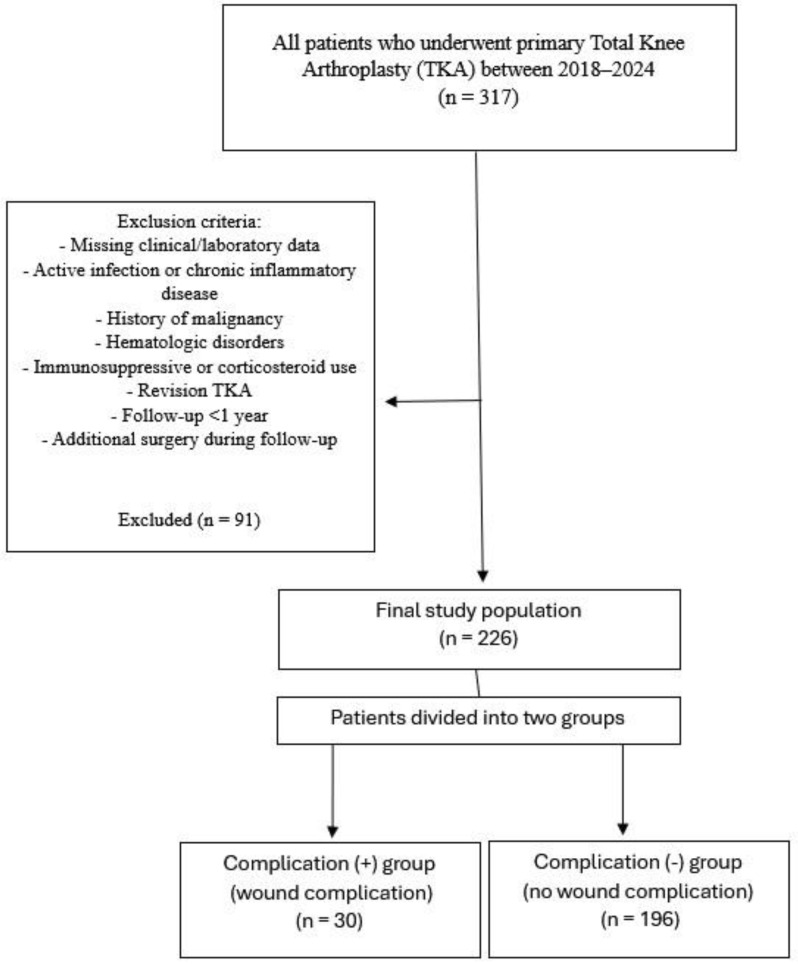
Flowchart illustrating the patient selection process and study design.

The hospital’s digital records scan revealed 317 patients who underwent total knee arthroplasty (TKA). After applying specific inclusion and exclusion criteria, 226 patients were included in the study (Figure 2).

**Figure 3:**
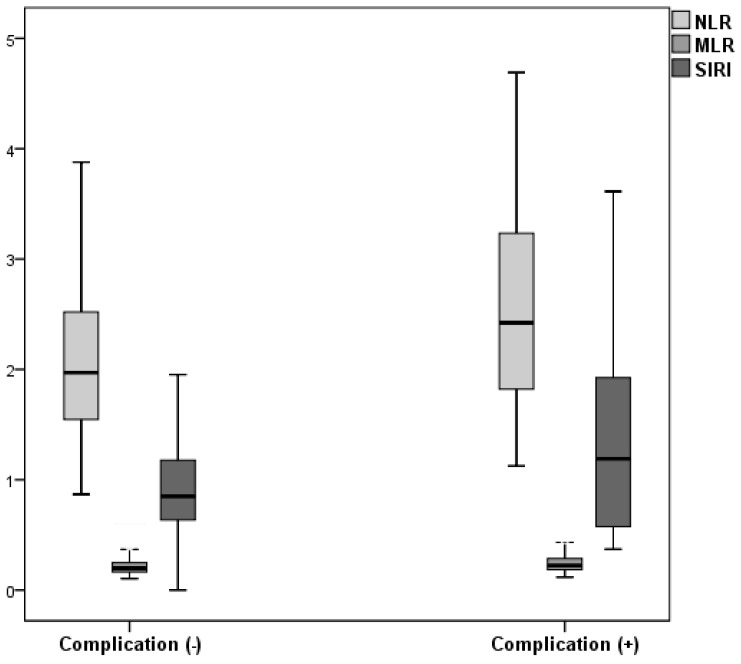
Error bar graphs representing the NLR, MLR, and SIRI values for complication (-) and complication (+) groups.

**Figure 4:**
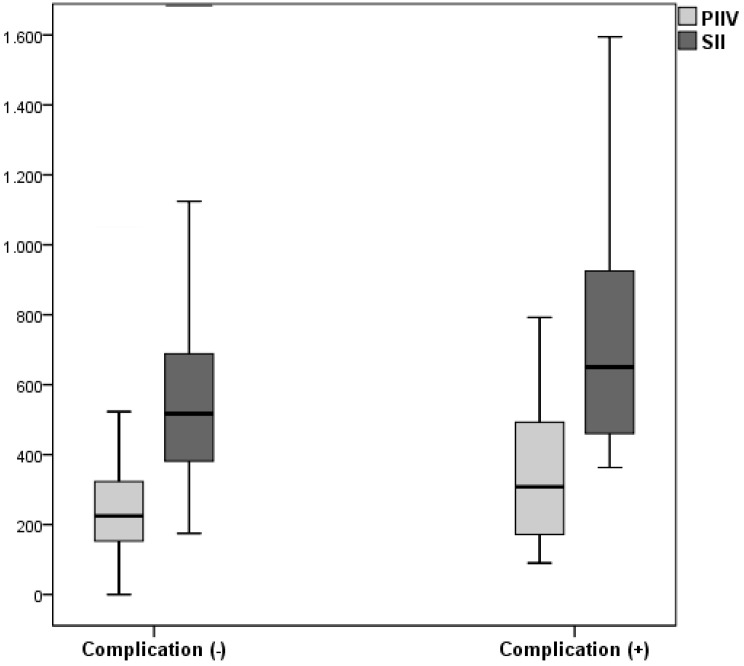
Error bar graphs representing the PIIV and SII values for complication (-) and complication (+) groups.

The demographic characteristics and biochemical data of the patients are presented in Table 1. No significant differences were found between the groups regarding age, gender, surgical side, or HbA1c, serum BUN, creatinine, total protein, albumin, and ALT levels (*p* > 0.05). However, the AST level was lower in the complication group (Z = −1.978, *p* = 0.048). On the other hand, the NLR (Z = −2.219, *p* = 0.026), MLR (Z = −1.965, *p* = 0.049), PIIV (Z = −2.213, *p* = 0.027), SII (Z = −2.240, *p* = 0.025), and SIRI (Z = −2.030, *p* = 0.042) values measured in the complication (+) group were higher than those in the complication (-) group (Table 1, Figure 3 and Figure 4).

The correlation analysis revealed a positive correlation between complication development and NLR (r = 0.148, *p* = 0.026), MLR (r = 0.131, *p* = 0.049), PIIV (r = 0.148, *p* = 0.027), SII (r = 0.149, *p* = 0.025), and SIRI (r = 0.135, *p* = 0.042). Conversely, a negative correlation was found between the AST level and complication development (r = −0.132, *p* = 0.048) (Table 2).

**Figure 5:**
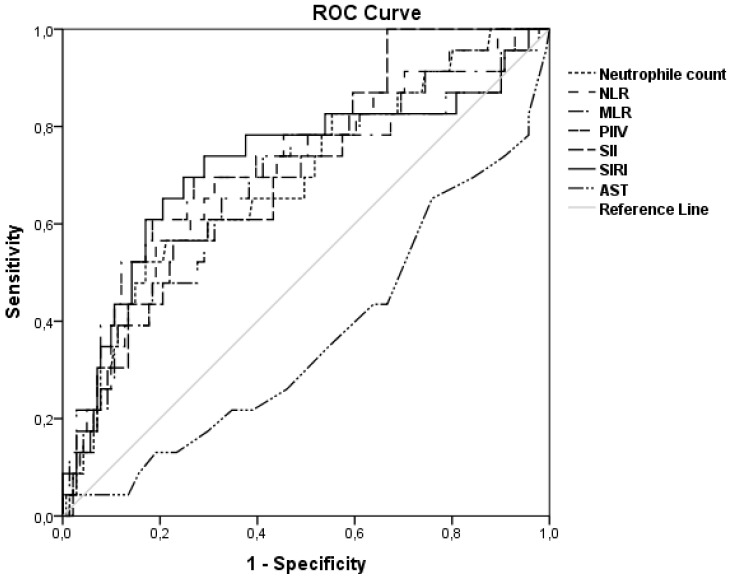
ROC curves for preoperative neutrophil count, NLR, MLR, PIIV, SII, SIRI, and AST values in predicting the risk of wound complications.

ROC curve analysis performed to identify hematological parameters predicting complication development showed that the risk of complications could be predicted by a neutrophil count > 4675 μL with 65% sensitivity and 61% specificity (area under the curve (AUC) = 0.689, *p* = 0.004, 95% confidence interval (CI) 0.570–0.809); an NLR > 2.35 with 70% sensitivity and 69% specificity (AUC = 0.709, *p* = 0.001, 95% CI 0.593–0.824); an MLR > 0.21 with 70% sensitivity and 62% specificity (AUC = 0.673, *p* = 0.008, 95% CI 0.543–0.804); a PIIV > 297 with 70% sensitivity and 73% specificity (AUC = 0.716, *p* = 0.001, 95% CI 0.589–0.842); an SII > 657 with 61% sensitivity and 70% specificity (AUC = 0.704, *p* = 0.002, 95% CI 0.597–0.810); and a SIRI > 1.17 with 70% sensitivity and 75% specificity (AUC = 0.720, *p* = 0.001, 95% CI 0.590–0.851) (Figure 5, Table 3).

Logistic regression analysis showed that the created model could predict the risk of complications with a high probability of 87%. Furthermore, a Hosmer–Lemeshow test was performed to test the significance of the model (X^2^ = 11.231, *p* = 0.189), and it was seen that the established logistic regression model was suitable for the data (the model worked well). The test was conducted using the “Backward Wald” option, and at the end of the test, it was found that among all parameters, only the parameter called SIRI was the best predictor of the risk of complications (B = 0.823, Wald = 8.124, *p* = 0.004, odds ratio = 2.277, 95% CI 1.293–4.010). These findings indicated that for every one-unit increase in the SIRI value above the threshold, the risk of developing complications more than doubles (Table 3).

To predict the risk of wound complications, a factor analysis was conducted to construct a scale entitled “Preoperative Inflammatory Index at Total Knee Arthroplasty (TKA-PII)” based on study parameters. The factor analysis conducted in this study yielded a Kaiser-Meyer-Olkin (KMO) test result of 0.61, indicating that the study population was adequate for analysis. Additionally, Bartlett’s Test of Sphericity, which yielded a value of 606.468, produced a *p*-value of less than 0.001, suggesting that the data were appropriate for factor analysis. The obtained eigenvalue was greater than 1, which helped identify the factors. The rotation matrix demonstrated that the study parameters clustered under a single factor. The results showed that the parameters “PIV,” “SII,” and “SIRI” each represent distinct factors and can be used to create a scale that predicts the risk of complications.

A reliability analysis was performed to assess the consistency of the scale. The reliability analysis conducted revealed a Hotelling’s T-Squared test result of 765.076 (*p* < 0.001), indicating a significant multivariate difference between the mean values of the groups. Additionally, the Cronbach’s alpha was found to be 0.65 (95% CI: 0.559–0.720). These results showed that this scale had a moderate-to-good reliability. Additionally, the F-test results demonstrated no significant similarity among the parameters forming this scale (F = 670.508, *p* < 0.001) (Table 4 and Table 5).

Gender-based analyses revealed that male patients had a significantly higher monocyte count (Z = −3.804, *p* < 0.001), with a parallel increase in MLR values (Z = −3.099, *p* = 0.002). Additionally, while serum creatinine (Z = −3.402, *p* = 0.001) and ALT (Z = −1.981, *p* = 0.048) levels were higher in males, their platelet count (Z = −2.605, *p* = 0.009), PLR (Z = −2.591, *p* = 0.010), and SII (Z = −2.594, *p* = 0.009) values were lower. Nevertheless, no significant gender differences were observed regarding the NLR, PIIV, and SIRI parameters (*p* > 0.05) (Table 6).

## 4. Discussion

Previous studies have reported that ratios such as NLR, MLR, and PLR, as well as composite indices like SII and SIRI, can help predict infection, thrombosis, and surgery-related complications [3,7,8,17]. Additionally, studies conducted in various clinical settings have demonstrated that indices like SII and SIRI are not only associated with infection but also with the length of hospital stay, the need for intensive care, and the overall clinical course of patients [18]. Additionally, studies related to orthopedic surgery have shown that inflammatory indices may be associated with postoperative clinical outcomes and that a high inflammatory burden can predispose patients to adverse events [3,19]. Moreover, studies examining periprosthetic joint infections following TKA have suggested that SIRI and SII values are elevated in infected patients, indicating that these parameters may possess diagnostic significance [19].

However, the literature also emphasizes that the standalone diagnostic accuracy of these indices may be limited, but their predictive power can increase significantly when combined with other inflammatory markers [20]. Similarly, studies investigating thromboembolic complications after total knee arthroplasty have reported that preoperative inflammatory indices are independent predictors and may represent a common biological basis for various types of complications [19]. These findings support the idea that postoperative complications are closely related not only to local factors but also to systemic predisposition to inflammation [21].

In the present study, although the neutrophil, lymphocyte, monocyte, and platelet counts were similar between the two groups, the NLR, MLR, PIIV, SII, and SIRI values were higher in the complication (+) group than in the complication (-) group. These findings suggested that the systemic inflammatory state may influence the postoperative healing process by reflecting not only the body’s response to surgery but also the patient’s preoperative biological “readiness level”.

The correlation analysis revealed a positive relationship between inflammatory indices and the development of complications. In addition, it was argued that preoperative neutrophil counts and NLR, MLR, PIIV, SII, and SIRI values could predict the risk of wound complications after surgery. Moreover, the SIRI value was the “best predictive marker” among the study parameters. This evidence suggests that systemic inflammation plays a significant role in wound healing. The underlying pathophysiology of this relationship can be understood through the bidirectional role of inflammation in the healing process. As is known, neutrophils are the primary effector cells in the early inflammatory response and react quickly to tissue damage. Subsequently, monocytes differentiate into macrophages, contributing to tissue repair and remodeling. Lymphocytes, on the other hand, play a critical role in regulating the inflammatory response. When the balance among these cell types is disrupted, it can lead to either excessive inflammation or insufficient immune regulation, both of which can adversely impact wound healing [10,22]. In this context, it was hypothesized that the systemic inflammatory burden present before surgical trauma could influence the severity of the sterile inflammatory response that develops postoperatively, ultimately affecting clinical outcomes.

On the other hand, the inflammatory response following orthopedic surgery is a dynamic process that undergoes significant changes, especially during the postoperative period. This response is characterized by an increase in neutrophils and a decrease in lymphocytes after surgical trauma, and it serves as a biochemical indicator of systemic inflammatory activity [23]. Indeed, some studies have indicated that even when preoperative values are similar, there are notable increases in inflammatory indices postoperatively, and these changes are linked to clinical outcomes [21,24]. Furthermore, the literature suggests that SIRI values can be associated with adverse outcomes in various clinical conditions, serving as a strong marker for patients’ prognosis [4,5,6,12,19,25]. In the present study, SIRI values were slightly higher in patients who developed wound complications compared to those who did not. Additionally, this parameter was the best potential marker for predicting the risk of wound complications. This suggests that SIRI, which combines neutrophil, monocyte, and lymphocyte counts, may provide better predictive performance than other indices in reflecting both the pro-inflammatory burden and the body’s capacity for immune regulation. By examining a broader range of non-infectious “wound complications”, the present study demonstrated that inflammatory indices could indicate not only the risk of infection but also the risk of complications associated with the overall healing process.

The results of this study indicated that the “TKA-PII” scale, which included the PIIV, SII, and SIRI parameters, was valid and reliable and could predict the risk of postoperative wound complications in patients undergoing TKA based on their preoperative inflammatory index data. Factor analysis revealed that these parameters are distinct and statistically independent. Subsequently, the threshold values of these parameters were presented on this scale, and each of these values was scored as 1 point. A score of zero indicates no risk of wound complications, while a score of three suggests a very high risk of developing complications. Although the reliability test results indicated that this scale had moderate validity and reliability, this study was preliminary, and its validity and reliability will be retested in a larger population.

### Limitations

This study had several limitations. First, the study was retrospective and single-center, carrying a potential risk of bias. However, from a clinical perspective, the most important aspect of our study is demonstrating that these indices, which can be obtained from routine complete blood counts in a homogeneous patient group without additional cost, can be integrated into the preoperative risk assessment process. In this regard, these indices stand out as more accessible, rapid, and inexpensive risk assessment tools compared to advanced molecular tests. Second, the study included only the measurement values of preoperative biochemical parameters, except C-reactive protein, erythrocyte sedimentation rate, and blood/urine/wound culture results. However, evaluating the results, it was argued that closer monitoring of patients, particularly those with high SIRI values, the implementation of perioperative optimization strategies, and more meticulous planning of wound care could contribute to the early prevention of potential postoperative complications. Nevertheless, it was also suggested that these indices are far from being the sole decision-makers and should be evaluated in conjunction with clinical risk factors. Third, because this study excluded patients with periprosthetic joint infection and those who underwent revision knee surgery, their preoperative blood biochemistry results, which can predict wound complications, were not identified. Finally, the patient’s nutritional status, which significantly impacts wound healing, was not addressed in this study. Despite these limitations, the results provide valuable insights for future research into hybrid biomarker models that assess both patient nutritional status and the acute inflammatory response together.

## 5. Conclusions

The study results demonstrate that preoperative NLR, MLR, PIIV, SII, and SIRI values could serve as independent predictive markers of wound complications following TKA. These indices, reflecting a multidimensional inflammatory response, have the potential to serve as biomarkers for integration into future clinical decision support systems. To address this potential need, the “TKA-PII” scale was developed in this study. The results showed that this scale could be a reliable and valid tool for predicting the risk of postoperative wound complications based on preoperative inflammatory index data in patients undergoing TKA. However, it was also argued that the validity and reliability of this test should be further supported by applying it to larger patient groups.

## Figures and Tables

**Table 1:** The table presents the comparative results of demographic findings and blood biochemical analyses between the patient groups. Mann–Whitney U test and Pearson’s chi-square test, *p* < 0.05.

		Complication (-)	Complication (+)		
Variables		Median (Min-Max)/ N (%)	Median (Min-Max)/ N (%)	Z/X^2^	*p*
Age (years)		68 (47–98)	68 (52–80)	−0.991 †	0.322
Sex	Female	166 (73.5%)	27 (11.9%)	0.587 ‡	0.443
	Male	30 (13.3%)	3 (1.3%)		
Site	Right	106 (46.9%)	14 (6.2%)	0.929 ‡	0.629
	Left	87 (38.5%)	15 (6.6%)		
	Bilateral	3 (1.3%)	1 (0.4%)		
Neutrophile (μL)		4470 (1900–10,190)	4570 (2100–9980)	−1.321 †	0.187
Lymphocyte (μL)		2115 (920–5160)	1900 (1050–5480)	−1.561 †	0.119
Monocyte (μL)		420 (180–3800)	460 (220–1100)	−0.739 †	0.460
Platelet (10^3^ μL)		269 (79–585)	281 (122–548)	−0.780 †	0.436
NLR		1.97 (0–6.82)	2.42 (1.13–4.69)	−2.219 †	0.026
MLR		0.20 (0.10–19.10)	0.23 (0.12–0.61)	−1.965 †	0.049
PLR		125.34 (40–501.09)	139.77 (40.33–365.33)	−1.855 †	0.064
PIIV		230.94 (0–1350)	307.94 (9.24–1228.11)	−2.213 †	0.027
SII		521.18 (174.61–3141.82)	650.63 (363.38–1594.94)	−2.240 †	0.025
SIRI		0.85 (0–3.39)	1.19 (0.37–3.61)	−2.030 †	0.042
BUN (mg/dL)		33 (17–91)	36 (19–66)	−1.425 †	0.154
Creatinine (mg/dL)		0.75 (0.46–1.61)	0.80 (0.60–1.51)	−1.866 †	0.062
HbA1C (g/dL)		6.20 (4.80–13.70)	6 (5.50–10.10)	−0.292 †	0.770
Protein (g/dL)		7.30 (4.40–8.40)	7.40 (4.80–8.17)	−0.965 †	0.335
Albumin (g/dL)		4.23 (2.90–5.20)	4.45 (2.96–5.00)	−1.865 †	0.062
ALT (U/L)		16 (5–51)	12.50 (7–43)	−1.442 †	0.149
AST (U/L)		18 (8–75)	15.50 (8–48)	−1.978 †	0.048

(†) value, Mann–Whitney U test; (‡) X2 value, Pearson Chi-square test; *p* < 0.05.

**Table 2:** This table outlines the study parameters that predict the risk of wound complications. ROC curve analysis and Logistic regression test, *p* < 0.05.

Variable		NLR	MLR	PIIV	SII	SIRI	AST
Complication risk	r	0.148	0.131	0.148	0.149	0.135	−0.132
	*p*	0.026	0.049	0.027	0.025	0.042	0.048

**Table 3:** This table shows the validity and reliability test results of the TKA-PII scale. Factor analysis and Reliability test, *p* < 0.05.

ROC-Curve Test for the Prediction of the Complication Risk							
						95% CI	
Variable	AUC	*p*	Cut-Off Value	Sensitivity	Specificity	Lower	Upper
Neutrophile	0.689	0.004	>4675 μL	65%	61%	0.570	0.809
NLR	0.709	0.001	>2.35	70%	69%	0.593	0.824
MLR	0.673	0.008	>0.21	70%	62%	0.543	0.804
PIIV	0.716	0.001	>297	70%	73%	0.589	0.842
SII	0.704	0.002	>657	61%	70%	0.597	0.810
SIRI	0.720	0.001	>1.17	70%	75%	0.590	0.851
**Logistic Regression Test for the Prediction of the Complication Risk**							
						**95% CI**	
**Variable**	**B**	***p***	**Wald**		**Odds Ratio**	**Lower**	**Upper**
SIRI	0.823	0.004	8.124		2.277	1.293	4.010

**Table 4:** This table details the parameters that constitute the TKA-PII scale.

	Factor Analysis Test					
	Factor 1 (Complication Risk)					
PIIV				0.92		
SII				0.99		
SIRI				0.72		
Eigenvalues				2.35		
Explained variance (%)				78.33		
Cumulative Variance (%)				78.33		
*Kaiser-Meyer-Olkin* test				0.609		
Bartlett’s test of Sphericity				606.468 (*p* < 0.001)		
	**Reliability Test**					
			**95% CI**		**F Test**	
**Variable**	**Cronbach’s** **Alpha**	**Intraclass** **Correlation**	**Lower** **Bound**	**Upper** **Bound**	**F**	***p***
TKA-PII scale	0.647	0.647	0.559	0.720	2.830	<0.001

Hotelling’s T-Squared test result of 765.076 (*p* < 0.001).

**Table 5:** This table compares the demographic data and blood biochemical analysis results between gender groups. Mann–Whitney U test and Pearson’s chi-square test, *p* < 0.05.

Parameter	Threshold Value	Score	Patient Score
PIIV *	>297	1	
SII **	>657	1	
SIRI ***	>1.17	1	
**Total score**			

(*) PIIV = (neutrophil count × monocyte count × platelet count)/lymphocyte count. (**) SII = (neutrophil count × platelet count)/lymphocyte count. (***) SIRI = (neutrophil count × monocyte count)/lymphocyte count.

**Table 6:** Sex-based comparison of demographic characteristics, laboratory findings, and inflammatory indices.

		Female	Male		
Variables		Median (Min-Max)/ N (%)	Median (Min-Max)/ N (%)	Z/X^2^	*p*
Age (years)		68 (47–98)	69 (55–84)	−1.330 †	0.184
Complication	No	166 (73.5%)	30 (13.3%)	0.587 ‡	0.443
	Yes	27 (11.9%)	3 (1.3%)		
Site	Right	106 (46.9%)	14 (6.2%)	5.197 ‡	0.074
	Left	85 (37.6%)	17 (7.5%)		
	Bilateral	2 (0.9%)	2 (0.9%)		
Neutrophile (μL)		4480 (1990–10,190)	4450 (1900–10,030)	−0.120 †	0.905
Lymphocyte (μL)		2110 (920–5480)	2110 (1080–3200)	−0.497 †	0.619
Monocyte (μL)		410 (180–1100)	530 (260–3800)	−3.804 †	<0.001
Platelet (10^3^ μL)		277 (79–585)	246 (110–537)	−2.605 †	0.009
NLR		2.00 (0.87–6.82)	2.04 (0–4.41)	−0.148 †	0.882
MLR		0.20 (0.10–0.61)	0.24 (0.16–19.10)	−3.099 †	0.002
PLR		128.13 (40.33–501.09)	104.27 (40–285.64)	−2.591 †	0.010
PIIV		243.60 (43.27–1350.98)	203.49 (0–1048.92)	−0.866 †	0.387
SII		562.88 (174.61–3141.82)	442.79 (188.42–1719.54)	−2.594 †	0.009
SIRI		0.85 (0.28–3.61)	0.98 (0–2.35)	−1.543 †	0.123
BUN (mg/dL)		33 (17–91)	33 (19–57)	−0.151 †	0.880
Creatinine (mg/dL)		0.75 (0.46–1.61)	0.87 (0.48–1.32)	−3.402 †	0.001
HbA1C (g/dL)		6.10 (4.80–13.70)	6.40 (4.80–10.10)	−0.932 †	0.351
Protein (g/dL)		4.29 (2.90–5)	4.37 (3.04–5.20)	−1.361 †	0.173
Albumin (g/dL)		7.30 (4.40–8.40)	7.40 (7.90–8.30)	−0.319 †	0.750
ALT (U/L)		15 (5–51)	17 (7–44)	−1.981 †	0.048
AST (U/L)		189 (8–75)	18 (10–40)	−1.813 †	0.070

(†) value, Mann–Whitney U test; (‡) X^2^ value, Pearson Chi-square test; *p* < 0.05.

## Data Availability

The data supporting the findings of this study are available from the corresponding author upon reasonable request.

## Abbreviations

The following abbreviations are used in this manuscript:

Min	Minimum
Max	Maximum
NLR	Neutrophil-to-lymphocyte ratio
MLR	Monocyte-to-lymphocyte ratio
PLR	Platelet-to-lymphocyte ratio
PIIV	Pan-immune-inflammation value
SII	Systemic immune-inflammation index
SIRI	Systemic inflammation response index
BUN	Blood urea nitrogen
HbA1C	Oxidized hemoglobin
ALT	Alanine aminotransferase
AST	Aspartate aminotransferase
